# Supplementary material for: Pyruvate Dehydrogenase Kinase 1 inhibition mediated oxidative phosphorylation enhancement in cartilage promotes osteoarthritis progression
Source: BMC Musculoskelet Disord. 2023 Jul 20;24:597. doi: 10.1186/s12891-023-06585-6 (PMC10357736; doi:10.1186/s12891-023-06585-6)
Supplement: Supplementary file 1 — Additional file 1. [file 12891_2023_6585_MOESM1_ESM.zip › MF.Gsea.1653627316242/GOMF_RECEPTOR_ANTAGONIST_ACTIVITY.html]

Details for gene set GOMF\_RECEPTOR\_ANTAGONIST\_ACTIVITY[GSEA]

|  || Dataset | 55457\_collapsed\_to\_symbols.cls.cls #OA\_versus\_normal.cls.cls #OA\_versus\_normal\_repos |
| Phenotype | cls.cls#OA\_versus\_normal\_repos |
| Upregulated in class | OA |
| GeneSet | GOMF\_RECEPTOR\_ANTAGONIST\_ACTIVITY |
| Enrichment Score (ES) | 0.37356004 |
| Normalized Enrichment Score (NES) | 1.157937 |
| Nominal p-value | 0.26117647 |
| FDR q-value | 0.6009968 |
| FWER p-Value | 1.0 |
Table: GSEA Results Summary

  

Fig 1: Enrichment plot: GOMF\_RECEPTOR\_ANTAGONIST\_ACTIVITY      
 Profile of the Running ES Score & Positions of GeneSet Members on the Rank Ordered List

  

| SYMBOL | TITLE | RANK IN GENE LIST | RANK METRIC SCORE | RUNNING ES | CORE ENRICHMENT || 1 | DKK2 | dickkopf WNT signaling pathway inhibitor 2 [Source:HGNC Symbol;Acc:HGNC:2892] | 33 | 1.033 | 0.1919 | Yes |
| 2 | LRPAP1 | LDL receptor related protein associated protein 1 [Source:HGNC Symbol;Acc:HGNC:6701] | 375 | 0.716 | 0.3004 | Yes |
| 3 | SPINT3 | "serine peptidase inhibitor, Kunitz type 3 [Source:HGNC Symbol;Acc:HGNC:11248]" | 809 | 0.567 | 0.3736 | Yes |
| 4 | DKKL1 | dickkopf like acrosomal protein 1 [Source:HGNC Symbol;Acc:HGNC:16528] | 3845 | 0.171 | 0.1703 | No |
| 5 | DKK3 | dickkopf WNT signaling pathway inhibitor 3 [Source:HGNC Symbol;Acc:HGNC:2893] | 5125 | 0.062 | 0.0827 | No |
| 6 | IGSF1 | immunoglobulin superfamily member 1 [Source:HGNC Symbol;Acc:HGNC:5948] | 5437 | 0.038 | 0.0657 | No |
| 7 | DKK1 | dickkopf WNT signaling pathway inhibitor 1 [Source:HGNC Symbol;Acc:HGNC:2891] | 6392 | -0.034 | -0.0019 | No |
| 8 | IL36RN | interleukin 36 receptor antagonist [Source:HGNC Symbol;Acc:HGNC:15561] | 6549 | -0.046 | -0.0055 | No |
| 9 | CCL5 | C-C motif chemokine ligand 5 [Source:HGNC Symbol;Acc:HGNC:10632] | 6584 | -0.048 | 0.0010 | No |
| 10 | PXDN | peroxidasin [Source:HGNC Symbol;Acc:HGNC:14966] | 7642 | -0.130 | -0.0566 | No |
| 11 | DKK4 | dickkopf WNT signaling pathway inhibitor 4 [Source:HGNC Symbol;Acc:HGNC:2894] | 8048 | -0.159 | -0.0581 | No |
| 12 | FST | follistatin [Source:HGNC Symbol;Acc:HGNC:3971] | 8511 | -0.194 | -0.0573 | No |
| 13 | ADH7 | "alcohol dehydrogenase 7 (class IV), mu or sigma polypeptide [Source:HGNC Symbol;Acc:HGNC:256]" | 8906 | -0.225 | -0.0455 | No |
| 14 | AGTR2 | angiotensin II receptor type 2 [Source:HGNC Symbol;Acc:HGNC:338] | 9009 | -0.232 | -0.0097 | No |
| 15 | IL1RN | interleukin 1 receptor antagonist [Source:HGNC Symbol;Acc:HGNC:6000] | 11014 | -0.432 | -0.0839 | No |
| 16 | ESR2 | estrogen receptor 2 [Source:HGNC Symbol;Acc:HGNC:3468] | 11595 | -0.515 | -0.0319 | No |
| 17 | IL18BP | interleukin 18 binding protein [Source:HGNC Symbol;Acc:HGNC:5987] | 12399 | -0.707 | 0.0389 | No |
Table: GSEA details [plain text format]

  

Fig 2: GOMF\_RECEPTOR\_ANTAGONIST\_ACTIVITY      
 Blue-Pink O' Gram in the Space of the Analyzed GeneSet

  

Fig 3: GOMF\_RECEPTOR\_ANTAGONIST\_ACTIVITY: Random ES distribution      
 Gene set null distribution of ES for **GOMF\_RECEPTOR\_ANTAGONIST\_ACTIVITY**
